# Volumetric Prefrontal Cortex Alterations in Patients With Alcohol Dependence and the Involvement of Self-Control

**DOI:** 10.1111/acer.14211

**Published:** 2019-11-05

**Authors:** Annika Rosenthal, Anne Beck, Evangelos Zois, Sabine Vollstädt-Klein, Henrik Walter, Falk Kiefer, Falk W. Lohoff, Katrin Charlet

**Affiliations:** Department of Psychiatry and Psychotherapy (AR, AB, HW, KC), Charité – Universitätsmedizin Berlin, Campus Mitte, Berlin, Germany; Department of Addictive Behavior and Addiction Medicine (EZ, FK), Medical Faculty Mannheim, Central Institute of Mental Health, University of Heidelberg, Heidelberg, Germany; and Section on Clinical Genomics and Experimental Therapeutics (CGET) (SV-K, FWL, KC), National Institutes of Health (NIH)/National Institute on Alcohol Abuse and Alcoholism (NIAAA), Bethesda, Maryland.

**Keywords:** Alcohol, Voxel-Based Morphometry, Sensation Seeking, Impulsivity, Prefrontal Cortex

## Abstract

**Background:**

Aspects of self-control such as sensation seeking and impaired impulse control have been implicated in alcohol dependence (ALC). Conversely, sensation seeking has been ascribed a possible protective role in stress-related psychopathologies. We therefore examined gray matter (GM) morphology in individuals with ALC, focusing on differences in prefrontal regions that have been associated with self-control. Additionally, we accounted for differences in lifetime alcohol intake regarding self-control measures and cortical structures in ALC patients.

**Methods:**

With voxel-based morphometry (VBM) focusing on prefrontal a priori defined regions of interest, we assessed a group of 62 detoxified ALC patients and 62 healthy controls (HC). ALC patients were subsequently divided into high (*n* = 9) and low consumers (*n* = 53). Self-control was assessed by use of the Barratt Impulsiveness Scale and the Sensation Seeking Scale.

**Results:**

Compared to HC, ALC had significantly less GM volume in bilateral middle frontal gyrus (MFG) and right medial prefrontal cortex as well as in the right anterior cingulate. High-consuming ALC showed smaller GM in right orbitofrontal cortex as well as lower sensation seeking scores than low consumers. In low-consuming ALC, right MFG-GM was positively associated with magnitude of sensation seeking; particularly, larger MFG-GM correlated with greater thrill and adventure seeking.

**Conclusion:**

Thus, our findings (i) indicate deficient GM volume in prefrontal areas related to self-control and (ii) might accentuate the phenotypic divergence of ALC patients and emphasize the importance of the development of individual treatment options.

ALCOHOL IS A psychoactive substance consumed by a significant part of the population worldwide (World Health Organization, 2014). In excess, alcohol use can have devastating consequences (Room et al., 2005; World Health Organization, 2014). The excessive harmful consumption of alcohol is one of the criteria of alcohol dependence (ALC) as defined by the Diagnostic and Statistical Manual of Mental Disorders (4th ed., text rev.; DSM–IV–TR; American Psychiatric Association, 2000). These facts emphasize the public and mental health concerns caused by this form of drug abuse.

With the aim of delineating the behavioral and biological components that might be involved in the reinforcement of substance use disorders, deficiencies in self-control have been identified as a contributing factor in the development of ALC (Bechara, 2005). By reflecting aspects of deteriorated self-control, impulsivity and sensation seeking, as defined by the affinity to explore novel and intense stimuli (Zuckerman, 2014), are hypothesized to be directly linked to the development and maintenance of substance use disorders such as ALC (Hittner and Swickert, 2006). Previous studies have linked the tendency to react prematurely and seek out risky situations with various aspects of the addiction process, including initiation of drug use, compulsive administration, and relapse (Dalley et al., 2011; Norbury and Husain, 2015). The lack of self-regulating ability has been ascribed to a shift of top-down cortical control to abundant striatal control (Goldstein and Volkow, 2011). Functional and structural changes in the mesocorticolimbic dopamine system, conceivably promoted by excessive alcohol intake (Charlet et al., 2013; Koob and Volkow, 2016), might enhance response to drug-associated stimuli in expense of successful self-control (e.g., Baler and Volkow, 2006). In line with that, reduced striatal dopamine D2 receptor (DRD2) availability and decreased dopaminergic synthesis correlated with craving and relapse susceptibility in alcohol-dependent subjects (Heinz et al., 1996, 2004). However, this DRD2 down-regulation is assumed to be counteradaptive, as no association with DRD2 genotype could be found (Heinz et al., 1995).

In consideration of the neuromodulatory effects of alcohol itself, drinking amount significantly varies within populations including individuals with ALC (Griswold et al., 2018; Wood et al., 2018), and furthermore, drinking pattern affects alcohol-attributable (health) consequences as well as treatment outcome and relapse (Miller et al., 2005; Rosoff et al., 2019; Wetterling et al., 1999). In particular, quantity of alcohol consumption differentially impacted gray matter (GM) structure in regions of the corticostriatal circuit (Charlet et al., 2014a,b; Yang et al., 2016) as well as thalamus (Segobin et al., 2014) in ALC patients. Therefore, it is warranted to consider severity levels of alcohol consumption in ALC, which might impact structural and functional deficits as previously shown (Charlet et al., 2014b; Guggenmos et al., 2018; Segobin et al., 2014; Yang et al., 2016).

Both impulsivity and sensation seeking have been associated with impairment of executive brain regions in the prefrontal cortex (PFC), which in turn has been repeatedly implicated to be dysfunctional in substance use disorders (Dalley et al., 2011). Functional and structural neuroimaging has proven valuable in identifying specific brain areas that seem to be predominantly involved in the inhibition of premature reaction such as the inferior frontal gyrus (IFG), the medial prefrontal cortex (mPFC), and the orbitofrontal cortex (OFC; Whelan et al., 2012; Wiers et al., 2015). Among prefrontal areas involved in self-regulation, activation and structure of middle frontal gyrus (MFG) as well as anterior cingulate gyrus (ACC) have been further linked to both sensation seeking and impulsivity (Dalley et al., 2011; Matsuo et al., 2009).

Higher trait impulsiveness was associated with smaller OFC volume in alcohol-naive adolescents before even initiation of alcohol consumption, whereas integrity of these adolescents’ OFC volumes was linked with good performance in perceptual reasoning, which is crucial in successful self-control (Schilling et al., 2013). Further, low self-control in childhood and adolescence predicted adult substance use disorders (Lee et al., 2011). Thus, the importance of accounting for anatomical alterations could prove as reliable indicators of vulnerability to develop addiction disorders with impulsive and sensation-seeking behavior. In contrast to that, as pointed out above, decreased behavioral self-control is ultimately influenced by the (neuro-adaptive) effects of excessive alcohol intake.

Previous studies have found moderate correlation between sensation seeking and impulsivity measures but conclude them to be distinct from one another (Ersche et al., 2010; Magid et al., 2007). In line with that, it has been proposed that both constructs differentially affect motives of alcohol consumption. While sensation seeking mediates alcohol use through enhancement incentives, impulsivity and consumption were linked through coping motives (Magid et al., 2007). Interestingly, sensation seeking has also been ascribed a possible protective feature in stress-related psychopathologies and also it supports handling of unknown, risky environments (Norbury and Husain, 2015). Animal models show resilience in novelty seeking rats exposed to stress (Driscoll et al., 1998). Furthermore, high sensation seeking has been hypothesized to be protective to the effects of stressful life events in adolescents (Smith et al., 1992). Stress reactivity has been shown to play a major role in the course of addiction, craving in particular (Volkow et al., 2016). Taking this into consideration, sensation seeking might factor into resilience against problems of substance abuse.

Based on differentiating subscales of sensation seeking, Zuckerman (1994) proposed an impulsive, unsocialized form comprised of, for example, Disinhibition and Experience Seeking, while Thrill and Adventure Seeking was denoted to be nonimpulsive and socialized (Zuckerman, 1994). This type of sensation seekers might seek out stimulating activities such as adventurous sports or professions limiting risk of drug abuse (Hansen and Breivik, 2001; Roberti, 2004).

Thus, in addition to the possible protective role of sensation seeking in ALC, it might potentially provide distinct exploratory capacity from impulsivity (Ersche et al., 2010).

Thus, results of structural imaging studies on sensation seeking and impulsivity have been inconclusive so far, and studies have been particularly conducted in healthy populations unaffected by substance abuse (Kwon et al., 2014; Matsuo et al., 2009; Tschernegg et al., 2015). Therefore, the aim of this study is the analysis of structural differences between healthy participants and inpatients who suffer from ALC in order to test previous findings that indicate the importance of self-control in the pathology of ALC by application of behavioral and psychometric measures of impulsivity and sensation seeking. Furthermore, we will examine the neurobiological underpinnings, focusing on structural differences in prefrontal regions that have been hypothesized to exert top-down control and self-regulatory functions that are assumed to be specifically dysfunctional in ALC patients. We further test for differences in lifetime alcohol intake regarding self-control measures and cortical structures in ALC patients in order to explore the potentially protective nature of sensation seeking.

## MATERIALS AND METHODS

### Participants

A total of 62 recently detoxified ALC patients and 62 healthy control (HC) subjects participated in the study protocol, which was approved by the local ethics committee. Subjects were matched by age and sex according to propensity scores with nearest-neighbor matching (d’Agostino, 1998). In addition, IQ, as measured by a vocabulary test (Schmidt and Metzler, 1992), was used as a matching variable.

HC were recruited through online advertisement. Treatment-seeking ALC patients were mainly recruited during inpatient treatment at Charité—Universitätsmedizin Berlin, Germany, as well as other collaborating hospitals in Berlin, as part of the National Genome Research Network (NGFN-Plus)/eMed program Alcohol Addiction of the SysMedAlcoholism consortium (Spanagel et al., 2013). All subjects were aged between 18 and 65 years and were right-handed according to Edinburgh Handedness Inventory (Old-field, 1971).

After receiving a study description in verbal and written form, all participants provided informed written consent according to the Declaration of Helsinki. Eligibility criteria for study participation in the ALC group were a diagnosis of alcohol dependence according to DSM-IV criteria [Diagnostic and Statistical Manual of Mental Disorders, Fourth Edition (DSM-IV), as well as completion of medically supervised detoxification. ALC were detoxified for 4 to 25 days (mean 12.55, SD 5.8) before MRI. In healthy controls (HC), the Alcohol Use Disorders Identification Test (AUDIT) was used to exclude control subjects who have suffered from alcohol abuse or ALC in the past 12 months (Saunders et al., 1993). In both groups, the amount of lifetime alcohol intake was determined by the use of the Lifetime Drinking History (LDH; Skinner and Sheu, 1982). Moreover, smoking behavior was measured by individual pack-years [sum of number of consumed cigarettes/18 (number of cigarettes per standard pack) × years of smoking phase] (see Charlet et al., 2014a). In addition, socioeconomic status (SES) was determined by educational degree level (Collins, 2016).

Exclusion criteria for all participants were Axis I disorders according to DSM-IV except for alcohol as well as nicotine abuse or dependence in ALC patients and nicotine abuse or dependence in hC (SCID-I; First et al., 1992). Furthermore, positive results in urine screenings for an extensive array of drugs that included THC, opiates, benzodiazepines, cocaine, or amphetamines led to exclusion of the subject. In addition, recent use of any psychotropic as well as anticonvulsive medication in ALC patients, claustrophobia, epilepsy, and other neurological or severe medical illnesses as well as pregnancy served as exclusion criteria. To assure safety according to the rules and regulations of the MRI scanner facilities, all participants with irremovable metal and metal containing tattoos or makeup were excluded from the study.

### Self-Control Measures

The *Barratt Impulsiveness Scale* (BIS 11) is a questionnaire used to evaluate various dimensions of impulsivity (Patton and Stanford, 1995). The BIS is separated into 3 subscores that include attention, motor, and nonplanning impulsiveness. A total BIS score was calculated and used in further analysis as well. High scores on all BIS scales respectively indicate higher levels of impulsivity. The reliability and validity of the BIS-11 as a measure of impulsivity in a range of domains are well established and documented (Stanford et al., 2009).

We used the updated form V of the *Sensation Seeking Scale* (SSSV). This scale consists of 30 self-report items measuring 3 of 4 questionnaire components: Thrill and Adventure Seeking (SSS-TAS), which assesses anticipation in high stimulus activities; Inhibition, which assesses participating in riskier activities; and Experience, which assesses participation in less risky situations. High scores on all SSS scales respectively indicate higher levels of sensation seeking. SSS total score was used to measure sensation seeking as an overall score (Zuckerman et al., 1964).

The *Lifetime Alcohol Drinking History* (LDH) is a structured interview in which the participants are asked about different patterns of their alcohol consumption from the beginning of regular alcohol intake up to the present. Accordingly, regular and maximum alcohol intake (amount/frequency) was recorded to calculate total lifetime alcohol consumption in kilograms of pure alcohol (*kg*). Ultimately, this provided a quantitative and clinical measure on each subject’s alcohol consumption (Skinner and Sheu, 1982).

The *Alcohol Dependence Scale* (ADS) generates a quantitative index of the severity of alcohol dependence including aspects such as impaired control over alcohol use, salience of drink-seeking behavior, tolerance, withdrawal symptoms, and a compulsive drinking style (Ross et al., 1990).

Additionally, we assessed the *Obsessive Compulsive Drinking Scale* (OCDS), which is a self-rating instrument that measures cognitive aspects of alcohol craving (Anton et al., 1996).

### Structural MRI

#### Structural MRI Acquisition.

Structural neuroimaging was conducted using 3T whole-body tomography (MAGNETOM Trio; Siemens, Germany). For each participant, a high-resolution T1-weighted anatomical image was acquired with a 3-dimensional magnetization-prepared rapid gradient-echo sequence: TR = 2.3 seconds, TE = 3.03 milliseconds, flip angle = 9°, slice thickness = 1 mm, voxel size = 1 × 1 × 1 mm, FoV = 256, and matrix = 256 × 256. Subjects were instructed to lay still during the MRI scanning procedure to minimize motion-induced data quality loss.

#### MRI Preprocessing.

Structural images were preprocessed and analyzed with SPM12 software (Wellcome Department of Cognitive Neurology, London), a software package designed to run within MATLAB version 7.8.0 (The MathWorks, Natick, MA).

MRI images of niftii format were analyzed by employing CAT 12 (Computational Anatomy Toolbox) to implement voxel-based morphometry (VBM) by Gaser and Dahnke ( http://www.neuro.uni-jena.de/vbm/). The software is an integrated toolbox allowing for completely automatic analyses of tissue class volumes that works within SPM12.

We used CAT 12 to for tissue class segmentation to extract GM, white matter (WM), and cerebrospinal fluid tissue class files. For spatial normalization, we employed DARTEL registration procedures. This procedure creates a mean of all images, which is then used as an initial template. Deformations from this template to each of the individual images are computed, and the template is then regenerated by applying the inverses of the deformations to the images. Normalization to the DARTEL template space resulted in a voxel size of 1.5 mm isotropic resolution. Data quality was checked via proof of sample homogeneity as well as through quality parameters that were estimated and saved as report files by CAT 12. These quality checks as well as an additional visual inspection led to inclusion of all 124 participants’ images.

Lastly, the images from the AD and healthy subjects were smoothed with an 8 mm FWHM isotropic Gaussian smoothing kernel via the SPM module “Smooth.” After the smoothing process, the voxels contain the averaged GM amount of all voxels in proximity. The range of this proximity is defined by the smoothing kernel. To accurately estimate GM volume differences in cortical regions, 8 mm FWHM has been shown to be superior to other kernel sizes (Chanraud et al., 2007).

Before statistical analysis, total intracranial volume (TIV) was automatically estimated by CAT 12 in order to be used as a covariate to correct for different brain sizes of the subjects.

#### Imaging Data Analyses.

A general linear model was estimated with the existing data to assess between-group differences in GM only. To account for confounding factors, differences in whole-brain GM as well as GM volume in regions of interest (ROIs) between groups were accounted for TIV, age, and gender by entering as covariates of no interest into the ANCOVA design matrix. As the group statistics showed highly significant differences between HC and ALC in terms of educational degree level, we subsequently reanalyzed all significant findings with the inclusion of this measure as a covariate. Because inclusion of pack-years as a covariate yielded equal results to its omission, we excluded the variable from our statistical models. Psychological and alcohol dependence measures were correlated with prefrontal GM by means of multiple regression.

#### Regions of Interest.

Based on previous studies (e.g., Whelan et al., 2012), we focused our analyses on fundamental brain regions associated with aspects of self-control: A priori ROIs for explicit masking and small-volume corrections were generated from the probabilistic *Neuromorphometrics atlas* ( http://www.neuromorphometrics.com/) by employing a MATLAB script. Single, unilateral anatomical labeling masks were created for (i) MFG, (ii) IFG, (iii) OFC, (iv) ACC, and (v) mPFC and subsequently merged into a large-scale ROI with the *ImCalc* function in SPM 12 (see Fig. S1 for detailed depiction of the a priori ROIs). For all ROI comparisons, small-volume correction at a threshold of *p <* 0.05 random-field theory (RFT)–based FWE (familywise error rate) adjustment was performed. This included adjustments for GM search volume to a spatial extent of 30 voxels to avert significant results attributed to noisy signals. Peak voxels that were significantly different between groups are reported in Montreal Neurological Institute (MNI) template coordinates. The peak voxel volumes of ROIs that displayed a significant between-group difference were extracted and entered into SPSS for further analyses. Following ROI analysis, a whole-brain exploratory analysis with a statistical threshold set at FWE-corrected *p <* 0.05 with a minimal cluster size of 30 voxels was conducted to investigate further GM differences throughout all regions.

### Statistical Analyses

Statistical analyses were conducted with SPSS software (version 23; SPSS Inc., Chicago, IL). Data were analyzed with independent-samples *t*-tests (*p <* 0.05) as well as bivariate or partial correlation analyses using Pearson correlations controlling for TIV, age, and sex. Again, since inclusion of pack-years did not change any results, we refrained from adding this variable into further analyses.

SPSS k-means clustering algorithm was applied to the LDH scale data in order to stratify patients into 2 groups based on their alcohol consumption. Two initial cluster centers were chosen, and the algorithm iteratively refined them by assigning each instance to its closest cluster center and updating this center to be the mean of its constituent instances (Jain, 2010).

Thus, we compared 53 low-consuming ALC (mean 520.4 kg lifetime alcohol intake) with 9 high-consuming ALC (mean 2097.7 kg lifetime alcohol intake). See Table S1 and Fig. S2 for descriptive details and the distribution of the groups.

For post hoc analyses, SPM eigenvariates were extracted for partial correlations and reported at a Bonferroni-corrected threshold of *p <* 0.005 (*p =* 0.05/10 Region of Interest) accounting for multiple testing.

We performed an additional exploratory mediation analysis to test whether LDH mediates the relationship between right MFG-GM volume and SSSV using PROCESS, a macro implemented in SPSS (Hayes, 2017). The tool generates a bias-corrected 95% bootstrap confidence interval to test for indirect effects of variables through a mediator.

## RESULTS

### Participants’ Characteristics

Characteristics of the study participants are displayed in Table 1. Both groups differed significantly regarding previous lifetime alcohol consumption, smoking, and affective scales. In detail, the ALC group consumed significantly more alcohol and nicotine over the course of a lifetime (see Table 1).

Bivariate correlations divided by HC and ALC show that there was small intercorrelation between measures of impulsivity and sensation seeking in both HC and ALC. Notably, in HC AUDIT score was positively correlated with SSSV Disinhibition (*r* = 0.35, *p* = 0.005) as well as BIS attentional impulsivity (*r* = 0.30, *p* = 0.018). In ALC, we found a negative correlation between LDH and SSSV Experience Seeking (*r* = −0.26, *p* = 0.04) and SSSV Thrill and Adventure Seeking (*r* = −0.31, *p* = 0.013). Moreover, BIS attentional impulsivity positively related to both ADS (*r* = 0.35, *p* = 0.006) and OCDS (*r* = 0.27, *p* = 0.024).

As expected, regarding Impulsivity and Sensation Seeking in ALC and HC, the ALC group displayed significantly higher scores in attentional impulsivity, nonplanning, and total BIS score than the control group (Table 2).

Furthermore, analyses of the subdivided ALC groups revealed significant differences in total SSSV as well as the Thrill and Adventure Seeking subscale, where higher sensation seeking was observed in ALC patients with lower lifetime alcohol consumption compared to ALC with high lifetime alcohol intake (Table 3). Finally, none of the subscale scores of the SSSV were correlated with impulsivity BIS scores when controlling for age and sex. Further, not ADS but OCDS scores differed between consumption groups (ADS; *p =* 0.571; OCDS; *p =* 0.011).

### VBM Group Comparisons

The optimized CAT 12 VBM ROI analysis detected significant between-group GM volume differences (Table 4). Specifically, GM ANCOVA analysis showed that ALC patients displayed lower local volumes in the left and right MFG as well as in the right ACC, and in the right mPFC compared to HC (see Table 4 for details). Inclusion of educational degree level did not change these significant group differences. ALC showed no greater GM volumes in a priori ROIs compared to HC. Further, comparing ALC with low alcohol consumption with ALC with high consumption did not reveal any significant GM differences. For results of whole-brain VBM analysis, see Fig. S3 and Table S2.

### VBM Correlations

**Fig. 1.**
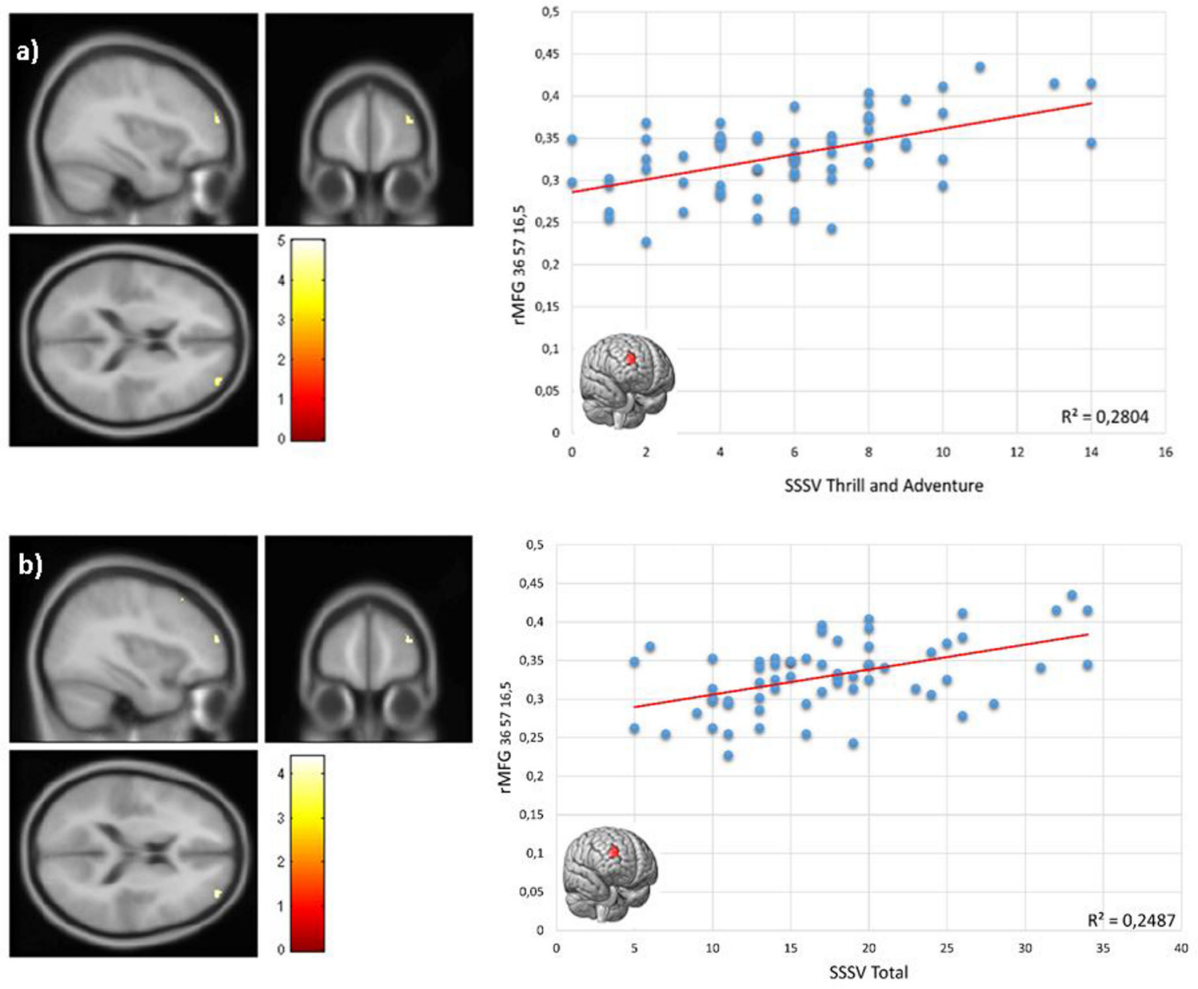
Results of multiple regression analyses. (**A**) In ALC patients, right MFG was positively correlated with SSSV Thrill and Adventure Score. (**B**) Right MFG was positively correlated with SSSV Total Score. The significance threshold was set at FWE-corrected *p* < 0.05. Color scales represent *t*-scores.

Multiple regression analyses indicated a significant positive correlation between right lateralized MFG-GM volume and sensation seeking as displayed by SSSV Total *(r =* 0.49, *R*^2^ *=* 0.249; *p*_FWE_ = 0.05) and SSSV Thrill and Adventure Seeking (*r* = 0.53, *R*^2^ = 0.280; *p*_FWE_ = 0.002) in the ALC group only (Fig. 1). When adjusting for the influence of educational degree level, right MFG and SSSV Thrill and Adventure Seeking remained a significant correlation (*p*_FWE_ = 0.009), while the relation between right MFG and total SSSV score was reduced to borderline significance (*p*_FWE_ = 0.051). Furthermore, subsequent analyses showed that the positive correlation between right MFG and SSSV Thrill and Adventure Seeking was significant (*p*_Bonferroni-corrected_ = 0.003) in the low-consumption group but failed to reach statistical significance in high-consuming ALC patients (*p*_Bonferroni-corrected_ = 1.00).

Regression of BIS scores on GM volume did not yield significant results for the ALC group or the healthy control group.

The additional exploratory mediation analysis did not reveal a significant LDH mediation of the association between right MFG-GM volume and SSSV Total (95% confidence interval 2.4075,19.4436) and SSSV Thrill and Adventure Seeking (95% confidence interval 0.2565, 10.0928), respectively.

Finally, our correlational analyses did not reveal significant associations between quantitative measures of alcohol use (lifetime drinking amount or duration of consumption) and GM volumes. However, a significant negative association between right OFC and ADS (*p*_FWE_ = 0.03; see Fig. S4) as well as a trend toward negative association between right OFC and OCDS (*p*_FWE_ = 0.07) was found in the ALC group. when including educational degree level into the analysis, the correlation diminished to a trend for ADS (*p*_FWE_ = 0.065). Post hoc analysis revealed that the associations between ADS and OFC were insignificant in the low-consumption group (*p*_Bonferroni-corrected_ = 0.1) and the high-consumption group (*p*_Bonferroni-corrected_ = 1.00). Results relating to OCDS were nonsignificant in the high-consumption group (*p*_Bonferroni-corrected_ = 1.0) and the low-consumption group (*p*_Bonferroni-corrected_ = 1.0), respectively.

## DISCUSSION

In this study, we assessed the relationship between aspects of self-control and alcohol dependence, primarily focusing on the structural brain correlates of impulsivity and sensation seeking in an ALC patient population compared to healthy volunteers. In addition, we also investigated neurobiological correlates of both self-control measures in low-versus high-consuming ALC patients and observed significantly different associations.

First, we found ALC subjects displaying significantly lower prefrontal cortical GM volume in nearly all areas of interest compared to healthy individuals. This validates the results of a vast amount of studies that found the PFC to be affected by structural volume loss in ALC (Heikkinen et al., 2017; Pfefferbaum et al., 1998; Sullivan et al., 2018).

However, no direct association between quantitative measures of alcohol intake and GM volumes was detectable. This could indicate that alcohol’s neuronal toxicity does not ultimately follow a simple linear dose-response trend in all ALC but rather needs to take other vulnerability factors into account too (e.g., different alcohol harm effects by different drinking levels and frequencies [cf. Charlet et al., 2013; Charlet and Heinz, 2017], or different neuromaturational processes [cf. Mashhoon et al., 2014]).

Noteworthy, we found a negative association between disease severity and right OFC in ALC. This is consistent with the results of previous structural MRI studies in ALC patients (e.g., Tanabe et al., 2009). Moreover, this region has been indicated to be dysfunctional in ALC populations negatively affecting motivation and emotion regulation (Volkow and Fowler, 2000), where smaller GM volume in OFC was further found to be associated with greater risk of relapse (e.g., Beck et al., 2012). We did not find significant relationships between disease severity and OFC in either the high- or low-consumption group, which might indicate a total group effect that cannot be explained by mere alcohol consumption.

Further, our results support the assumption that ALC patients exhibit higher self-reported impulsivity as indicated by the BIS scores. Interestingly, they did not differ from HC on any sensation seeking criterion. Findings of elevated trait impulsivity in ALC and other addictions are numerous (for a review, see Potenza and De Wit, 2010), and sensation seeking has been linked to ALC in relation to that (Hittner and Swickert, 2006). However, when partialing out the influence of sex and age in our sample, no intercorrelation between these 2 distinct measures was found. It is to consider that the constructs measure distinct dimensions of impulsivity (Ersche et al., 2010). In our ALC subgroups, we found that high-consuming ALC displayed significantly lower sensation seeking scores than ALC patients with less consumption.

Our analysis of structural brain correlates of sensation seeking revealed similar conjunct results. In the overall ALC group, a cluster in the right MFG was positively related to the Thrill and Adventure as well as total SSSV score. Further subgroup analyses revealed that the low-consuming ALC group drove this association, while no association was found in ALC with high lifetime alcohol intake. Commonly, sensation seeking is understood to display an aspect of loss of control, often characterized by lack of prefrontal function and GM loss (e.g., Holmes et al., 2016). As stated above, the thrill and SSSV is often termed as a socialized type of sensation seeking that is not characterized by impulsivity (De Brabander et al., 1996).

Our results might be in favor of this assumption because the SSSV Thrill and Adventure subscale was positively associated with the right MFG in our low-consuming ALC subgroup.

On the other hand, little is known about the effects of alcohol intake on sensation seeking itself. Most studies conclude that sensation seeking might be a risk factor for the initiation of problematic drinking, and a large part of research has been done in populations without ALC (Hittner and Swickert, 2006). Finally, the form in which sensation seeking and impulsivity can be expressed is highly dependent on environmental factors, too. Higher SES can provide more favorable outlets such as stimulating sports or travel, whereas a low socioeconomic background might offer riskier means of stimulation (Norbury and Husain, 2015). Inclusion of educational degree level as a proxy of SES did not change our primary results, but a construct of other measures such as income and work status should be considered in future studies.

In addition, a number of studies concluded that trait impulsivity is associated with dysfunctional prefrontal cortical regions that have been assumed to be reflected by reduction of GM volume and thickness (Matsuo et al., 2009). In contrast to that, studies like Cho and colleagues (2013) have found positive correlations between prefrontal GM volume and impulsivity measures. In line with that, the lack of our findings regarding structural correlates of BIS-assessed impulsivity is plausible, although we found the ALC group surpassed the HC group in terms of BIS scores. Again, environmental factors lack consideration in most of the research focusing on the structural correlates of impulsivity. As an example, serotonergic function influencing impulsive, aggressive, and drinking behavior has been shown to be modulated by early adverse experiences in monkeys (e.g., Heinz et al., 2003). Various studies in humans also point out the impact of early stressors that affect structural brain development (e.g., Moog et al., 2018).

There are some limitations to the generalization of our results. Firstly, our ALC sample included more male than female patients. However, because treatment-seeking ALC population seen in hospital is often overrepresentative of men, a study addressing specifically gender differences in the association of cortical structure and self-control phenotypes would need special diligence with regard to an even participants’ gender selection. Further, cross-sectional study designs do not disentangle questions of causality and directionality; future research could investigate particularly the question of predisposition by, for example, inclusion of unaffected first-degree relatives (Ersche et al., 2010), or by conducting longitudinally designed studies. In addition, alcohol consumption data collected by retrospective self-report may be vulnerable to the subjectivity inherent in the anamnestic method. Here, future studies need to replicate these findings using other types of sensation seeking or impulsivity assessment, respectively, such as behavioral measures in conjunction with participants rated by collaterals. Finally, the correlation between MFG and SSSV Thrill and Adventure Seeking was of intermediate effect size (Cohen’s *d* = 0.55). Stringent FWE correction and relatively small sample size (*n* = 62 in each group) might decrease the power of this study; this may particularly apply to our subgroup stratification (low- vs. high-consuming ALC) by *k*-means clustering, resulting in unequal groups. The exploratory findings in such a small subgroup are therefore of preliminary (cautious) nature and should be replicated in further studies. Moreover, future studies might therefore also include larger (balanced) samples. In addition, our structural analysis was restricted to a priori defined prefrontal regions, which did not allow us to explore differentiated effects in other areas of the brain.

In conclusion, our results show next to significantly lower prefrontal cortical GM volumes in our ALC group compared to the HC that treatment-seeking ALC patients consuming lower alcohol amounts (mean 520.4 kg of pure alcohol or lower) throughout lifetime compared to patients with higher amounts (mean 2097.7 kg of pure alcohol or higher) demonstrate more sensation seeking and greater right MFG-GM volume. In particular, the thrill and adventure seeking subcomponent was positively associated with right MFG-GM volume hinting at a possible protective role of thrill and adventure seeking in terms of lower alcohol consumption and preserved prefrontal brain structure. Thus, our findings (i) indicate deficient GM in prefrontal areas related to self-control and (ii) might accentuate the phenotypic divergence of ALC patients and emphasize the importance of the development of individual treatment options.

## Supplementary Material

supp info

## Figures and Tables

**Table 1.** Characteristics of Alcohol-Dependent Patients and Healthy Controls

	Alcohol- dependent patients (46 male/ 16 female)^a^		Healthy controls (39 male/23 female)^a^		
			
Descriptive data	Mean	SD	Mean	SD	*p*- value

Age (years)	44.56	11.18	42.74	11.69	0.37
IQ (vocabulary)	99.95	16.49	104.26	10.033	0.08
Educational degree level	2.45	0.65	2.05	0.69	>0.01^b^
LDH (lifetime alcohol consumption in kg)	784.86	759.24	58.75	80	>0.01^b^
Pack-years of cigarette consumption	24.36	24.73	10.17	13.57	>0.01^b^
AUDIT (sum score)	NA	NA	3.29	2.6	NA
TIV	1,536.43	140.17	1,571.14	148.01	0.182

AUDIT, Alcohol Use Disorders Identification Test; IQ, intelligence quotient (based on vocabulary test); LDH, Lifetime Drinking History; NA, not applicable; SD, standard deviation; TIV, total intracranial volume.

a Chi-square test showed no significant difference for gender between both groups (χ^2^ = 1.83, *p* = 0.176).

b Two-tailed independent-samples *t*-tests revealed a significant difference between groups (*p* < 0.05).

**Table 2.** Correlation Matrix of All Demographic and Psychological Variables Assessed: (a) ALC Group and (b) HC Group

	(a) Alcohol-dependent patients																
	
	1	2	3	4	5	6	7	8	9	10	11	12	13	14	15	16	17

1. Gender																	
2. Age	−0.11																
3. Educational degree level	−0.17	−0.04															
4. Lifetime drinking history	0.15	0.32*	−0.22														
5. BIS-11 attention	−0.16	−0.02	−0.08	0.18													
6. BIS-11 motor	−0.06	−0.16	0.48**	−0.11	0.04												
7. BIS-11 nonplanning	−0.05	−0.1	0.01	−0.15	0.35**	0.11											
8. BIS-11 total score	−0.13	−0.13	0.17	−0.05	0.68**	0.52**	0.79**										
9. SSSV disinhibition	0.08	−0.36**	0.2	−0.1	−0.06	0.17	0.2	0.16									
10. SSSV experience seeking	−0.2	−0.22	0.34**	−0.26*	−0.07	0.26*	0.26*	0.24	0.55**								
11. SSSV thrill and adventure seeking	0.13	−0.35**	0.11	−0.31*	−0.1	0.17	0.1	0.09	0.46**	0.62**							
12. SSSV total score	0.02	−0.38**	0.25	−0.27*	−0.09	0.24	0.22	0.19	0.80**	0.85*	0.86**						
13. ADS	.	−0.11	−0.15	0.26*	0.35**	−0.01	0.25	0.30*	0.05	0.02	0.06	0.05					
14. OCDS	0.04	0.06	−0.04	0.41**	0.29*	−0.09	−0.04	0.08	−0.15	−0.12	−0.25’	−0.21	0.51**				
15. TIV	0.33*	−0.15	−0.08	−0.09	0.30*	0.08	0.37**	0.38**	0.12	0.01	0.1	0.1	0.01	−0.08			
16. Pack-years	0.07	0.30*	−0.02	0.19	−0.12	−0.09	−0.23	−0.23	−0.15	−0.04	−0.11	−0.12	0.01	0.02	−0.21	
17. IQ	−0.13	0.11	0.38*	0.07	0.01	0.32*	−0.09	0.09	0.11	0.17	−0.11	0.05	0.01	−0.13	−0.01	0.11	

	(b) Healthy controls															
	
	1	2	3	4	5	6	7	8	9	10	11	12	13	14	15	16

1. Gender																
2. Age	0.01																
3. Educational degree level	0.23	−0.13														
4. Lifetime Drinking History	0.21	−0.13	0.27*													
5. AUDIT Total Score	0.24	−0.29*	0.18	0.40**												
6. BIS-11 Attention	−0.28*	−0.18	−0.1	0.09	0.30*											
7. BIS-11 Motor	0.04	−0.12	0.16	−0.14	0.01	−0.1										
8. BIS-11 Nonplanning	−0.32*	−0.06	−0.27*	0.03	0.16	0.63**	−0.01									
9. BIS-11 Total Score	−0.29*	−0.17	−0.13	−0.01	0.23	0.77**	0.39**	0.85**								
10. SSSV Disinhibition	0.1	−0.22	0.02	0.22	0.35**	0.04	0.27*	0.08	0.18							
11. SSSV Experience Seeking	−0.05	0.2	0.01	−0.03	−0.04	−0.11	0.2	−0.01	0.04	0.55**						
12. SSSV Thrill and Adventure Seeking	0.06	−0.16	0.13	0.05	0.01	−0.1	0.17	−0.1	−0.03	0.49**	0.55**					
13. SSSV Total Score	0.05	−0.09	0.07	0.11	0.14	−0.06	0.26*	−0.01	0.08	0.83**	0.82**	0.83**				
14. TIV	0.58**	−0.27*	0.13	0.01	0.30*	−0.09	0.27*	−0.11	0.02	0.24	0.11	0.2	0.23			
15. Pack-years	0.1	0.31*	−0.14	0.21	−0.03	0.09	−0.06	0.16	0.11	−0.09	0.06	−0.07	−0.05	−0.04		
16. IQ	0.39**	0.34**	0.43**	0.24	0.02	−0.13	0.13	−0.27*	−0.16	−0.08	0.05	−0.11	−0.06	0.04	0.16	

ADS, Alcohol Dependence Scale; BIS, Barrat Impulsiveness Scale; OCDS, Obsessive Compulsive Drinking Scale; SSSV, Sensation Seeking Scale Form V; TIV, total intracranial volume.

* Correlation is significant at the 0.05 level (2-tailed).

** Correlation is significant at the 0.01 level (2-tailed).

**Table 3.** Mean Values and SD of Impulsiveness and Sensation Seeking, and Between-Group Comparisons Using Independent-Group *t*-Tests

	Alcohol- dependent patients		Healthy controls			
				
	Mean	SD	Mean	SD	*t*-value	*p*-value

Impulsivity						
BIS A.I.	15.48	3.56	13.94	3.39	2.483	0.014^a^
BIS M.I.	21.74	3.39	22.03	3.38	−0.485	n.s.
BIS N.P.	26.56	4.45	24.05	4.24	3.225	0.002^a^
BIS total	63.74	7.67	60.02	7.46	2.912	0.007^a^
Sensation seeking						
SSSV-ES	6.84	2.519	7.31	2.556	−0.834	n.s.
SSSV-DIS	4.74	2.78	4.90	3.09	−0.306	n.s.
SSSV-TAS	5.84	3.25	6.77	3.09	1.646	n.s.
SSSV total	17.45	7.09	18.92	7.16	1.147	n.s.

A.I., Attention Impulsiveness; BIS, Barrat Impulsiveness Scale; DIS, Disinhibition; ES, Experience Seeking; M.I., Motor Impulsiveness; N.P., Nonplanning Impulsiveness; n.s., not significant; SSSV, Sensation Seeking Scale Form V; SD, standard deviation; TAS, Thrill and Adventure Seeking.

a Two-tailed independent-samples *t*-tests indicated significant group differences.

**Table 4.** Significant Differences in Sensation Seeking Subscale Scores and Total Score Between the ALC Subgroup With High and Low Lifetime Alcohol Consumption

	Low consumption		High consumption			
				
	Mean	SD	Mean	SD	*t*-value	*p*-value

Sensation seeking						
SSSV-ES	7.09	2.490	5.88	1.885	1.628	n.s
SSSV-DIS	4.83	2.914	4.38	1.847	0.594	n.s.
SSSV-TAS	6.30	3.196	2.88	2.031	4.072	0.001^a^
SSSV total	18.23	7.292	13.13	3.271	3.335	0.03^a^

DIS, Disinhibition; ES, Experience Seeking; n.s., not significant; SD, standard deviation; SSSV, Sensation Seeking Scale Form V; TAS, Thrill and Adventure Seeking.

a Two-Tailed Independent-Samples *t*-Tests Indicated Significant Group Differences.

**Table 5.** Gray Matter Volume Differences Found in Alcohol-Dependent Patients (ALC) Compared to Healthy Controls in Predefined Regions of Interest (ROIs)

				MNI coordinates		
				
Brain structure	*T*	*p*_FWE_	Cluster size (k)	*x*	*y*	*z*

Left MFG	6.5329	0.0000	252	−27	31.5	42
Right mPFC	5.6058	0.0002	96	1.5	51	−7.5
Right MFG	5.3315	0.0007	30	46.5	13.5	33
Right ACC	4.9942	0.0026	42	4.5	28.5	31.5

Results are derived from VBM-ANCOVA controlled for confounding effects of age, gender, and total intracranial volume. Results are reported at FWE-corrected *p* < 0.005.

ACC, anterior cingulate gyrus; FWE, familywise error; MFG, middle frontal gyrus; MNI, Montreal Neurological Institute; mPFC, medial prefrontal cortex; uncorr., uncorrected.
