# Supplementary material for: Volumetric Prefrontal Cortex Alterations in Patients With Alcohol Dependence and the Involvement of Self-Control
Source: Alcohol Clin Exp Res. Author manuscript; Available in PMC 2020 Dec 1. (PMC6904522; doi:10.1111/acer.14211)

**Figure S1** A Priori defined ROI masks based on the CAT12 DARTEL Neuromorphometrics Atlas (Gaser & Dahnke, Jena University Hospital, Departments of Psychiatry and Neurology).


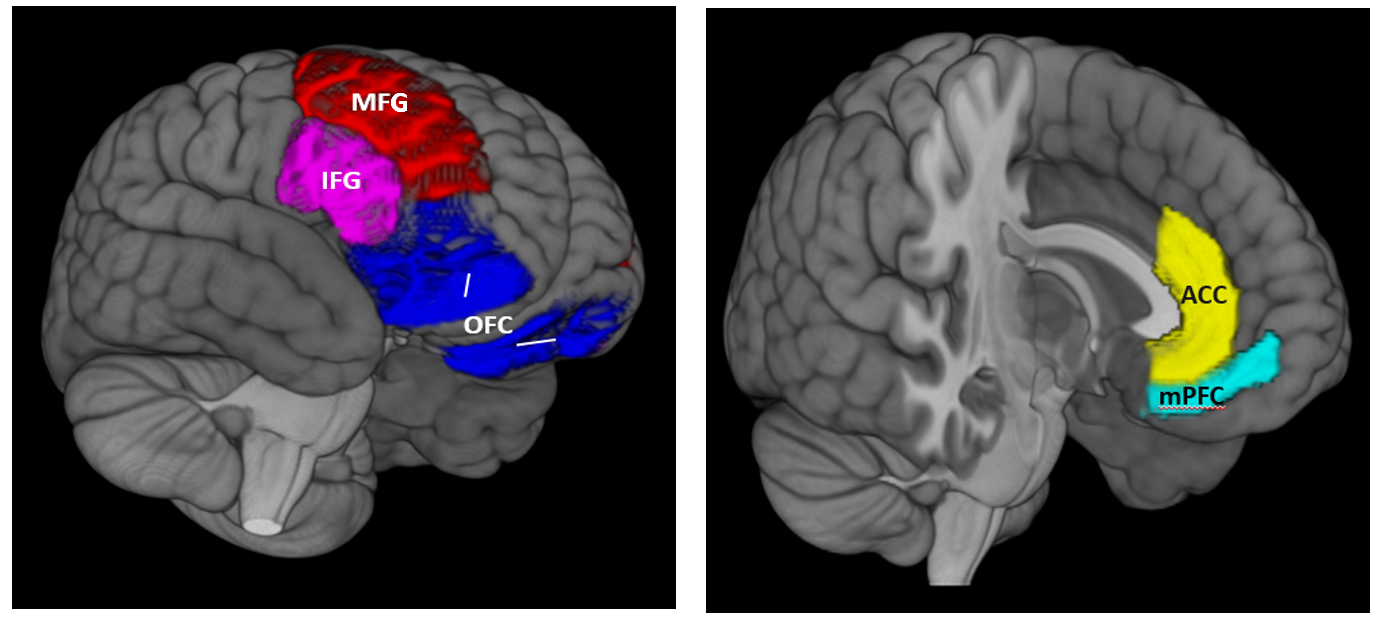


**Table S1** Significant differences in LDH scores and age between ALC subgroup with high and low lifetime alcohol consumption.

|  |  | ***Low Consumption***  *(38 male/15 female)* | | ***High Consumption***  *(8 male/ 1 female)* | |  |  |
| --- | --- | --- | --- | --- | --- | --- | --- |
|  |  | *Mean* | *SD* | *Mean* | *SD* | *t- value* | *p value* |
|  | *LDH* | *520398,89* | *267467,34* | *2097744,43* | *478630,41* | *-13,84* | *<0.001^a^* |
|  |  |  |  |  |  |  |  |
|  | *Age* | *43,64* | *11,68* | *49,25* | *5,09* | *-2,33* | *0.03* |
| *^a^Two-tailed independent samples t-Tests indicated significant group differences; SD = standard deviation; LDH = Lifetime Drinking History* | | | | | | | |
|  |  |  |  |  |  |  |  |
|  |  |  |  |  |  |  |  |

**Figure S2** Histogram of the distribution of the two ALC groups that were created by k-means clustering to categorize lifetime drinking amount into high and low consumption.

**
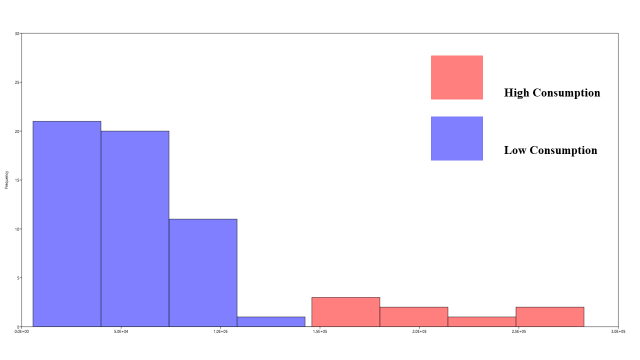
**

**Table S2** Whole-brain grey matter volume differences between alcohol-dependent (ALC) patients and healthy controls. Results are derived from VBM-ANCOVA controlled for confounding effects of age, gender and total intracranial volume (TIV). Results reported at p<.005 whole-brain FWE-corrected with a minimal cluster size of 30 voxels.

| Brain Structure | Clustersize kE | T (peak) | Z (peak) | p (FWE) | MNI coordinates | | |
| --- | --- | --- | --- | --- | --- | --- | --- |
|  |  |  |  |  | x,y,z | x,y,z | x,y,z |
| Right Medial Precentral Gyrus | 1896 | 6,61 | 6,09 | 0,0000 | 1,5 | -21 | 67,5 |
|  |  | 6,12 | 5,69 | 0,0001 | 1,5 | -24 | 48 |
|  |  | 5,91 | 5,52 | 0,0004 | 4,5 | -16,5 | 46,5 |
| Left Middle Frontal Gyrus | 339 | 6,53 | 6,03 | 0,0000 | -27 | 31,5 | 42 |
| Right Middle Temporal Gyrus | 1511 | 6,26 | 5,81 | 0,0001 | 52,5 | -21 | -12 |
|  |  | 6,20 | 5,76 | 0,0001 | 64,5 | -33 | -9 |
|  |  | 5,97 | 5,57 | 0,0003 | 57 | -10,5 | -12 |
| Left Medial Frontal Cortex | 333 | 6,17 | 5,73 | 0,0001 | 0 | 49,5 | -6 |
| Right Thalamus Propper | 332 | 5,97 | 5,57 | 0,0003 | 16,5 | -33 | 0 |
| Left Superior Temporal Gyrus | 200 | 5,55 | 5,22 | 0,0016 | -57 | -1,5 | 0 |
| Right Supramarginal Gyrus | 357 | 5,55 | 5,22 | 0,0016 | 54 | -40,5 | 49,5 |
|  |  | 5,25 | 4,97 | 0,0053 | 51 | -48 | 43,5 |
|  |  | 4,89 | 4,66 | 0,0206 | 52,5 | -49,5 | 33 |
| Left Third Ventricle | 413 | 5,50 | 5,18 | 0,0020 | 0 | -6 | -1,5 |
|  |  | 5,36 | 5,06 | 0,0034 | -1,5 | -7,5 | 9 |
| Left Hippocampus | 123 | 5,38 | 5,08 | 0,0031 | -15 | -37,5 | 1,5 |
| Right Middle Frontal Gyrus | 30 | 5,33 | 5,04 | 0,0039 | 46,5 | 13,5 | 33 |
| Right Exterior Cerebellum | 235 | 5,31 | 5,02 | 0,0043 | 34,5 | -58,5 | -28,5 |
|  | 159 | 5,29 | 5,00 | 0,0046 | 1,5 | 4,5 | -1,5 |
| Right Superior Frontal Gyrus | 96 | 5,23 | 4,95 | 0,0058 | 18 | 61,5 | 19,5 |
| Left Superior Frontal Gyrus | 35 | 4,98 | 4,73 | 0,0152 | -25,5 | -7,5 | 55,5 |
| Left Fusiform Gyrus | 62 | 4,97 | 4,73 | 0,0154 | -22,5 | -60 | -12 |
| Left Fusiform Gyrus | 30 | 4,94 | 4,70 | 0,0176 | -33 | -39 | -21 |
| Right Gyrus Rectus | 42 | 4,93 | 4,70 | 0,0178 | 3 | 48 | -21 |
| Right Exterior Cerebellum | 52 | 4,81 | 4,59 | 0,0278 | 19,5 | -51 | -13,5 |

| kE = expected voxels per cluster; FWE = family-wise error; MNI = Montreal Neurological Institute |
| --- |

**Figure S3** Whole brain Grey Matter Differences. The results of an exploratory FWE-corrected VBM analysis ALC < healthy controls rendered onto the CH2better template in MRIcroGL. *For detailed results: See table S2.*


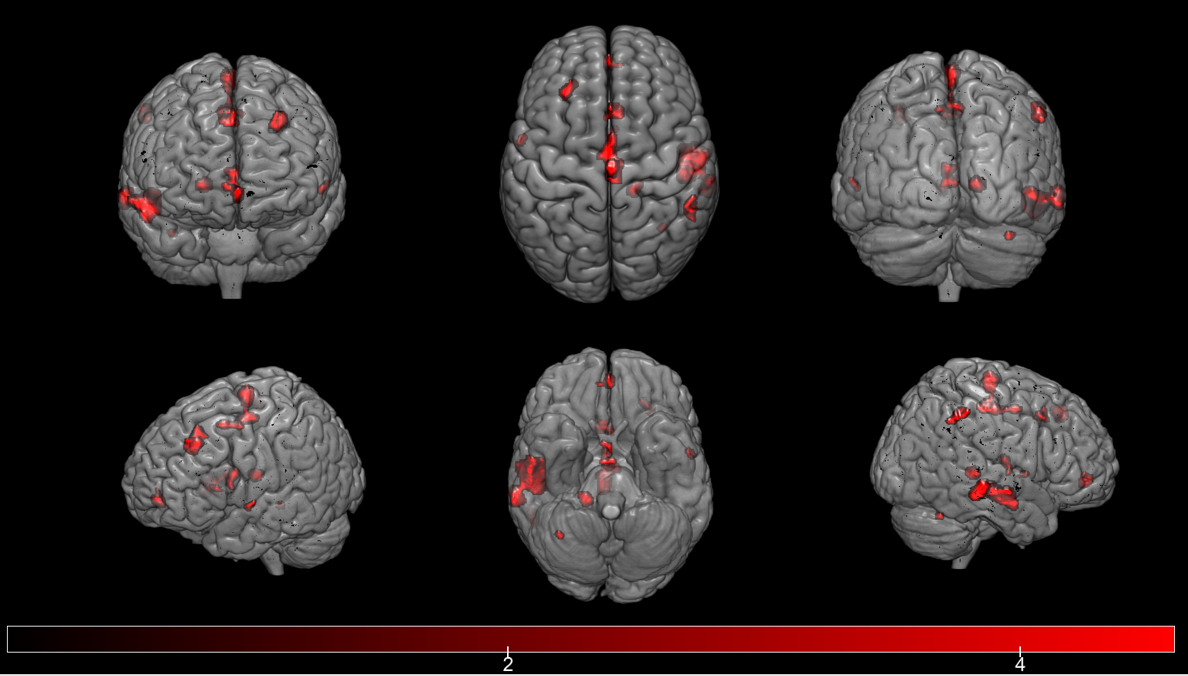


**Figure S4** Results of multiple regression analyses. a) In ALC patients right OFC was inversely correlated with ADS score.


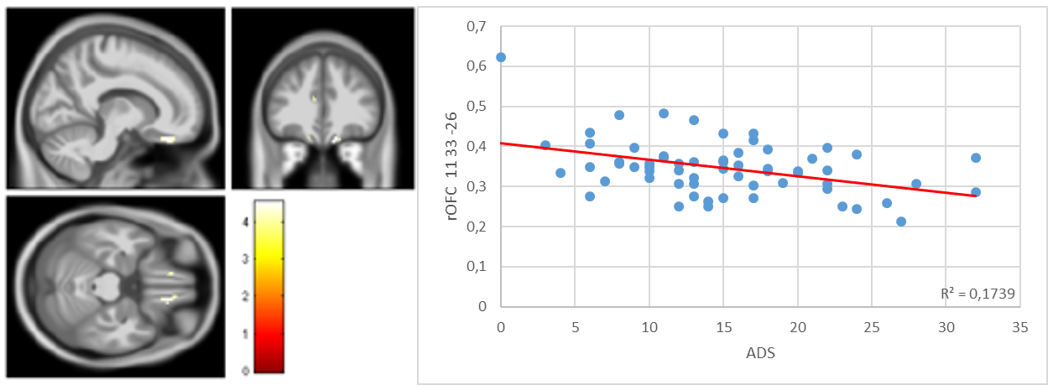

Supplement: supp info [file NIHMS1054066-supplement-supp_info.docx]
